# Genotypic diversity and unrecognized antifungal resistance among populations of *Candida glabrata* from positive blood cultures

**DOI:** 10.21203/rs.3.rs-2706400/v1

**Published:** 2023-04-03

**Authors:** Hassan Badrane, Shaoji Cheng, Christopher L Dupont, Binghua Hao, Eileen Driscoll, Kristin Morder, Guojun Liu, Anthony Newbrough, Giuseppe Fleres, Drishti Kaul, Josh L Espinoza, Cornelius J Clancy, M. Hong Nguyen

**Affiliations:** 1 University of Pittsburgh, Pittsburgh, Pennsylvania, USA;; 2 J. Craig Venter Institute, La Jolla, CA 92037,; 3 VA Pittsburgh Healthcare System, Pittsburgh, Pennsylvania, USA

## Abstract

The longstanding paradigm is that most bloodstream infections (BSIs) are caused by a single organism. We performed whole genome sequencing of five-to-ten strains from blood culture (BC) bottles in each of ten patients with *Candida glabrata* BSI. We demonstrated that BCs contained mixed populations of clonal but genetically diverse strains. Genetically distinct strains from two patients exhibited phenotypes that were potentially important during BSIs, including differences in susceptibility to antifungal agents and phagocytosis. In both patients, the clinical microbiology lab recovered a fluconazole-susceptible index strain, but we identified mixed fluconazole-susceptible and –resistant populations. Diversity in drug susceptibility was likely clinically relevant, as fluconazole-resistant strains were subsequently recovered by the clinical laboratory during persistent or relapsing infections. In one patient, unrecognized respiration-deficient small colony variants were fluconazole-resistant and significantly attenuated for virulence during murine candidiasis. Our data suggest a new population-based paradigm of *C. glabrata* genotypic and phenotypic diversity during BSIs.

## INTRODUCTION

*Candida* spp. are remarkably versatile opportunistic pathogens capable of colonizing or causing invasive infections of diverse anatomical sites. Candidemia is the most common type of invasive candidiasis and the fourth leading bloodstream infection (BSI) in the United States^1,2^. *Candida glabrata*, the second most prevalent agent of invasive candidiasis, is notable among *Candida* spp. for its haploid, rather than diploid genome, and its propensity to develop antifungal resistance. Like most Candida, *C. glabrata* are human gastrointestinal (GI) tract commensals. In the face of disturbed homeostasis, as in persons who are critically ill, immunosuppressed, receiving broad spectrum antibiotics, undergoing GI surgery or suffering from disruptions of mucosal integrity, *C. glabrata* can invade the bloodstream and other deep tissues. Candida strains often manifest striking genomic plasticity, which is believed to facilitate adaptation and survival under diverse and changing conditions^3–10^. Whole genome sequencing (WGSing) of longitudinal *Candida* strains from humans or infected mice often reveal substantial genomic differences, including point mutations, insertions, deletions, and whole or partial chromosomal aneuploidies^7^.

The longstanding paradigm is that most BSIs, including candidemia, are caused by a population of genetically identical strains derived from a single organism that passes through a bottleneck (“single organism” or “independent action” hypothesis).^11,12^ Standard microbiology laboratory protocol in processing positive clinical cultures is to test single microbial strains from morphologically distinct colonies. Increasingly, WGS data demonstrate that colonization or chronic infections by various bacteria may be caused by a population of strains in which genetic diversity emerges during long-term host interactions^13–15^. At present, it is unknown if BSIs are commonly caused by genetically diverse populations, rather than single organisms. We recently demonstrated, for the first time, that positive blood cultures from individual patients with monomicrobial bacterial BSI (carbapenem-resistant *Klebsiella pneumoniae*) were comprised of mixed populations of genetically and phenotypically diverse strains, including those demonstrating differences in antibiotic susceptibility and virulence^16^.

In this study, we tested the hypothesis that contemporaneous *C. glabrata* strains from individual patients with BSIs were genetically and phenotypically diverse. We evaluated *C. glabrata* BSIs in 10 patients. For each patient, the clinical lab isolated an index strain from a single colony morphotype. We analyzed WGSs of the index and 4–9 other strains recovered from independent colonies. We then assessed phenotypes of genetically distinct strains from 2 patients. As hypothesized, we detected within-patient genotypic and phenotypic diversity, including antifungal resistance, that was not recognized by the clinical laboratory.

## Results.

### Patients with *C. glabrata* BSIs.

We enrolled 10 patients with *C. glabrata* BSIs [Table 1]. Nine patients had underlying diseases of the GI or biliary tracts (exception: patient H). Two patients had breakthrough *C. glabrata* BSIs while receiving fluconazole (patients I, J), and 3 others received an antifungal agent in the 3 months preceding BSI (fluconazole (patients H and L), caspofungin (patient D)). In one patient, the index *C. glabrata* strain (i.e., strain isolated by the clinical microbiology lab) was fluconazole-resistant (patient EF); index strains from the other 9 patients were fluconazole-susceptible-dose dependent (S-DD). Four patients died within 30 days of *C. glabrata* BSI diagnosis (patients D, K, L, P).

### WGS of *C. glabrata* strains.

**Figure 1.**
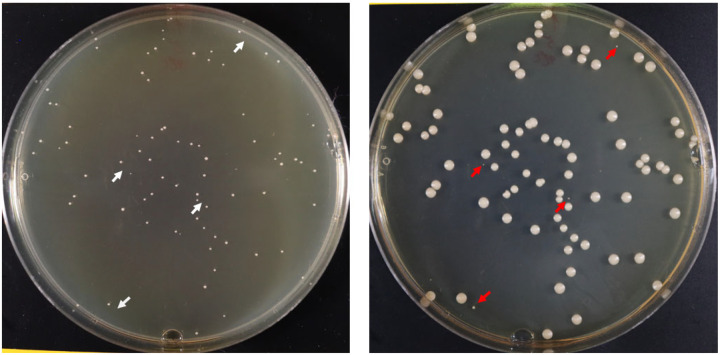
Morphology of *C. glabrata* strains recovered from the positive blood culture of patient J. Ten microliters from the blood culture bottle were plated onto Sabouraud Dextrose agar plates and incubated at 35°C. Plates shown on the left and right are from 48 and 84 hours of incubation, respectively. Note that smaller colonies were not visible at 48 hours (white arrows), and first became evident at 84 hours (red arrows).

We streaked 10 μL aliquots from the initial positive blood culture bottle from each of the 10 patients onto SDA plates, which were incubated at 35°C for 48 hours. For 9 patients, colonies were indistinguishable by morphotypes. We isolated strains from 4 to 9 from randomly selected colonies. In the tenth patient (patient J), colonies were indistinguishable by morphotypes at 48 and 72 hours of incubation. At 84 hours, however, pinpoint colonies were evident, admixed with a greater number of larger colonies [Figure 1]. For the remainder of the manuscript, we refer the larger and pinpoint colonies as normal (NCV) and small colony variants (SCV), respectively. We isolated 4 NCV and 5 SCV strains for further study. The index J strain grew as a NCV. For each patient, the index strain was labeled as strain #1, and other independently isolated strains were labeled as #2–10 (#2–5 for patient I). In patient J, strains #1–5 and #6–10 were NCV and SCV, respectively, on SDA plates. All BSI strains underwent next generation sequencing (Illumina NextSeq). Index strains also underwent sequencing by Oxford Nanopore (ONT) using MinION.

Strains had 13 chromosomes and closed genomes of 12.7–12.98 Mbp. The closed genome of reference strain *C. glabrata* CBS138 is 12.34 Mbp. On average, 98% of reads from clinical *C. glabrata* were mapped to the reference genome (range: 96%−99%). Single nucleotide polymorphisms (SNPs) within-patient were not distributed uniformly along the chromosomes, but rather were predominantly clustered in hot spots, particularly in subtelomeric regions [Supplemental Figure 1].

### Genotypic diversity of *C. glabrata* strains.

#### Nucleotide differences and genome variants.

**Figure 2.**
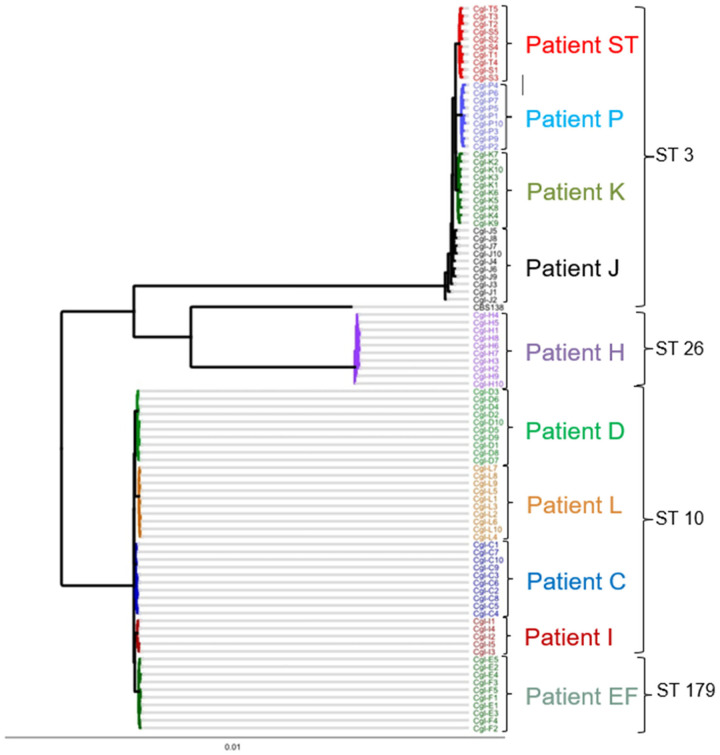

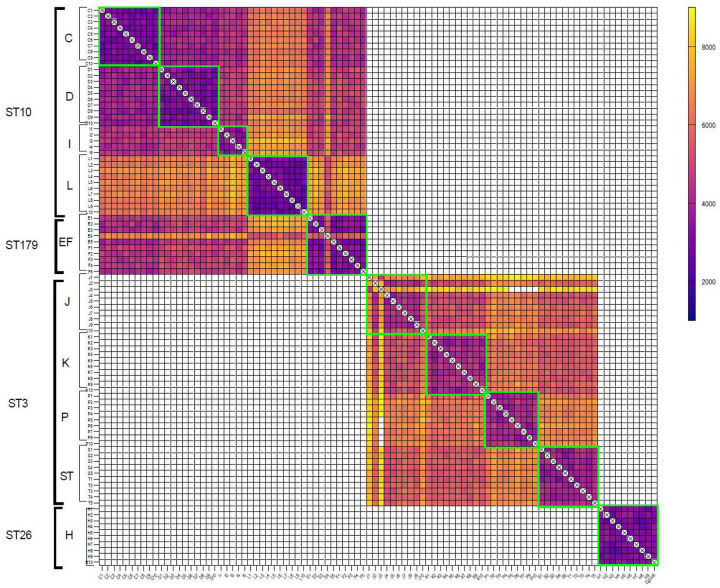

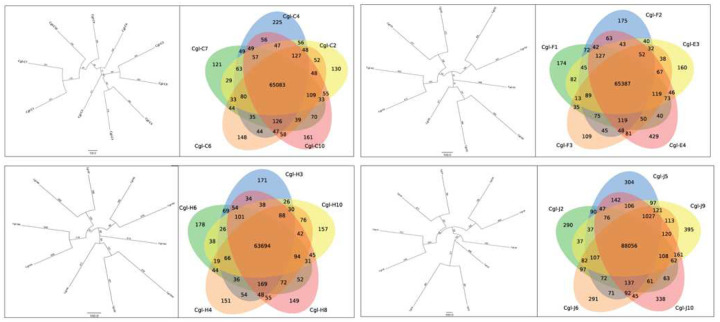
Phylogenetic analysis of *C. glabrata* strains from blood cultures of patients with bloodstream infections. **2A**. Phylogenomic tree of the 94 BSI strains and reference strain CBS138, as estimated using Maximum Likelihood with RaxML. It is based on a genome alignment generated based on variant calling data. Within-patient diversity is demonstrated for all strains, in each patient. Strains from a given patient are color coded. ST: sequence type. **2B**. Whole genome heat map showing the pairwise SNP distances of the intra- and inter-patient *C. glabrata* strains recovered from blood culture bottles. Strains from a given patient are bracketed and labeled with the appropriate letter. Strains are grouped by sequence type (ST), as indicated. Strains from patient J showed the greatest within-patient diversity. **2C**. Venn diagrams of numbers of genome variants, comparing 4 *C. glabrata* strains from a representative patient of each clade.

To estimate genetic relationships among the 94 *C. glabrata* strains, we built a high-resolution phylogenetic tree based on nucleotide differences (SNPs or insertions-deletions (indels)), estimated by comparison with *C. glabrata* CBS138 [Figures 2A and B]. Overall, SNPs and indels accounted for 89% and 11% of nucleotide differences, respectively. Strains represented 4 sequence types (STs), and they segregated into three distinct clades: 1) ST3 (patients J, K, P, ST), 2) ST26 (patient H), 3) ST10 (patients C, D, I, L), and ST179 (patient EF) [Figures 2A and B]. Within clades, strains clustered by patient. Nodes grouping within-clade and within-patient strains had high bootstrap support values (≥85). Deduced nucleotide sequence percent identity ranged from 99.02% to 99.98% for pairwise comparison of all clinical isolates. Despite this genetic relatedness, within-patient strains were clearly divergent, based on unique SNPs and/or indels. There was a within-patient average of 3,598 nucleotide differences across all 10 patients. J and C strains showed the highest (7,507) and lowest (2,067) within-patient nucleotide differences, respectively.

Results were similar when strains were compared by number of genome variants (i.e., unique genome sites at which SNPs or indels were observed). Representative Venn diagrams for within-patient genome variant comparisons are shown in Figure 2C. On average, 70.4% and 29.6% of variants were synonymous and non-synonymous, respectively; 99.5% and 0.5% of the non-synonymous SNPs were missense and nonsense, respectively.

Using a genome assembly and alignment approach with a Nextflow pipeline, we identified within-patient gene copy number variants (CNVs) in 4 patients (patients EF, J, H, and K). Twelve genes were deleted among strains from these patients, 8 of which encoded adhesins [Table 2]. There were no within-patient gene duplications or large-scale genetic differences such as aneuploidies.

Finally, we analyzed variants associated with non-synonymous mutations that discriminated within-patient strains (i.e., mutations found in at least one strain from a patient but not found in all strains, compared to *C. glabrata* CBS138). Gene Ontology (GO) term analysis revealed a significant over-representation of genes encoding adhesins and cell wall proteins [Table 3]. J strains were also significantly enriched for non-synonymous variants in genes involved in mitochondrial functions.

#### Mitochondrial genome differences.

**Figure 3.**
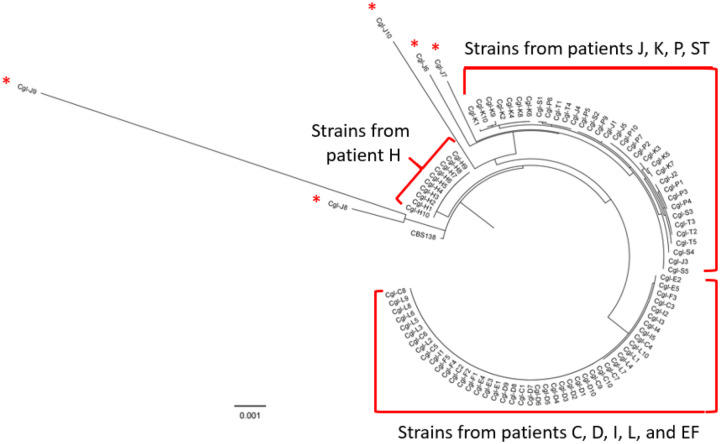

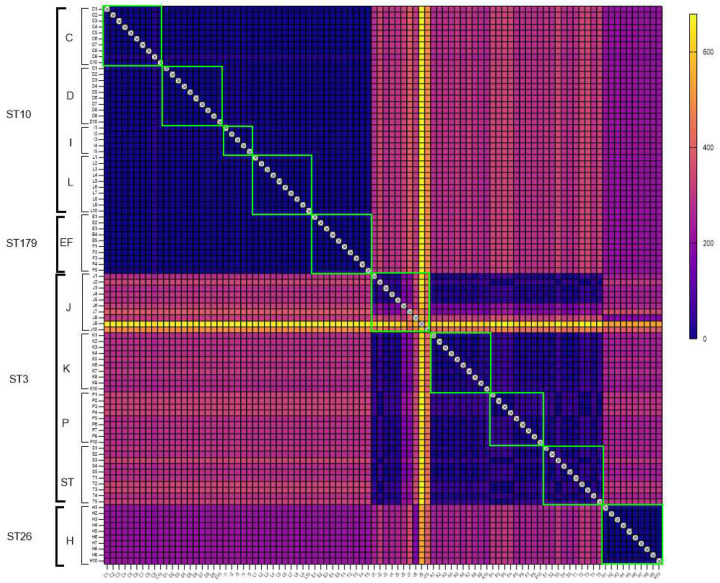

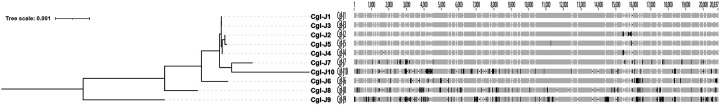
Mitochondrial genome phylogeny of *C. glabrata* strains from blood cultures of patients with bloodstream infections. **3A**. Phylogenomic tree for all strains, estimated using Maximum Likelihood with RaxML. The tree represents mitochondrial genome nucleotide alignment of the 94 strains, based on variant calling data. We used 100 iteration bootstrapping, and only values higher than 50 are shown. Strains J1-J5 carried fewer mitochondrial nucleotide differences than did J6-J10, and they clustered in a clade with K, P and ST strains, in keeping with nuclear phylogeny. Strains J6-J10 (red asterisks) showed much higher divergence than did J1-J5, and fall outside of the clade that include strains J1-J5. **3B. Heat map of pairwise mitochondrial genome nucleotide differences**. Strains are grouped by sequence type (ST), as indicated. Strains from patient J showed unusually high within-patient diversity, indeed the highest diversity among all 94 isolates. **3C. High resolution mitochondrial genome phylogeny of 10 *C. glabrata* strains from patient J**. Alignment graph of mitochondrial genomes from the 10 patient strains (right), and corresponding phylogeny (left). Coordinates of nucleotide position are shown on top of alignment. Grey colored positions represent nucleotides identical to the consensus. Black vertical bars show presence of SNPs or insertions, and black horizontal lines show deletions. Phylogeny was estimated using RaxML.

We constructed a phylogenetic tree using maximum likelihood based on nucleotide differences in mitochondrial genomes among the 94 BSI *C. glabrata* strains [Figure 3A]. Mitochondrial genome homology (i.e., DNA identity) among strains from patient J was 96.71%−99.98%. For other strains, within-patient homology was higher (99.69%−100% DNA identity). Overall, the 94 *C. glabrata* strains were segregated by mitochondrial phylogeny into the same clades as with nuclear phylogeny [Figures 3A]. H strains again constituted their own clade. Within the other 2 clades, strains did not cluster cleanly by patient due to very high DNA identity. [Figure 3A]. Strains J1-J5 carried fewer mitochondrial nucleotide differences than did J6-J10 [Figure 3B and 3C], and they clustered in a clade with P, K and ST strains, in keeping with nuclear phylogeny. Strains J6-J10 showed much higher divergence (DNA identity: 96.71%−99.21%) than did J1-J5 (99.71% - 99.98%).

#### Summary.

We demonstrated genotypic diversity among *C. glabrata* strains recovered from positive blood cultures in each of 10 patients.

### Phenotypic diversity of *C. glabrata* strains.

We evaluated whether certain genotypically distinct strains manifested differences in clinically relevant phenotypes. We studied strains from patients L and J in greater detail because genomic data suggested within-patient differences that might be associated with antifungal resistance that was not recognized by the clinical microbiology laboratory.

#### Patient L.

Upon diagnosis of fluconazole-susceptible *C. glabrata* BSI, patient L was treated with IV fluconazole. Blood cultures ~48 hours later remained positive for *C. glabrata*, which was now identified by the clinical microbiology laboratory as azole-resistant [Table 1].

Strains L1-L10 were similar in colony size and morphology on YPD and RPMI agar plates, and in growth rates in YPD broth at 37°C. Genome variants that discriminated between L strains (i.e., mutations found in at least one strain, but not in all 10 strains) were noted in 53 genes encoding adhesins [Supplemental Table 2]. We assessed adherence of representative strains L2, L4 and L6 to Hep-2 cells *in vitro*. These strains harbored mutations in 22, 30 and 30 adhesin genes, respectively. Three, eight and five adhesin genes had mutations in the respective strains, without being mutated in the other 2 strains. Adherence of strain L2 to Hep-2 cells was significantly higher (91.6±9.2%) than that of either L4 or L6 (66.3±3.4% and 78.0±1.7%, respectively; p=0.03, Brown-Forsythe ANOVA test); the latter strains did not significantly differ from each other in adherence.

**Figure 4.**
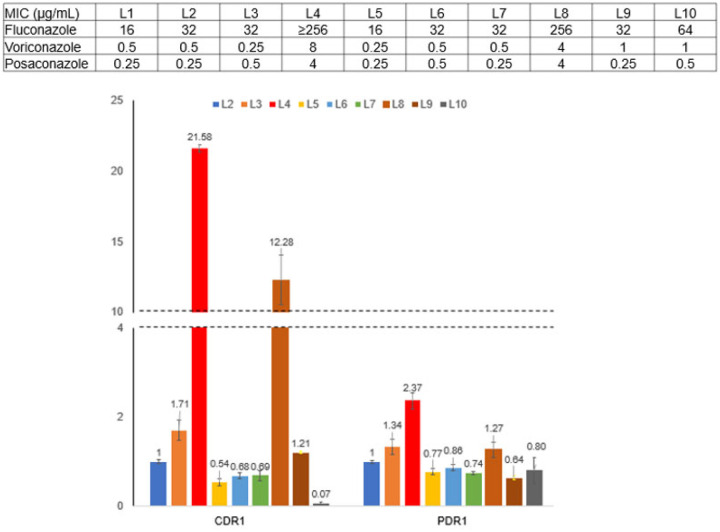
Azole susceptibility and *PDR1* and *CDR1* expression by *C. glabrata* strains from patient L. Strains L4 and L8 were resistant to fluconazole compared with the other 8 BSI L strains. By RT-PCR, *CDR1* was over-expressed in strains L4 and L8 compared with other L strains. *PDR1* was over-expressed in L4 compared to other strains. Data presented are median and interquartile range from triplicate experiments.

All strains except L4 and L8 exhibited antifungal MICs similar to those of the index strain. Fluconazole, posaconazole and isavuconazole MICs against strain L4 were ≥8-fold, ≥16-fold and ≥32-fold higher, respectively, than those against other L strains [Figure 4]. L4 carried a mutation within *PDR1*, conferring a G346C substitution within the central regulatory domain of transcriptional regulator Pdr1.^17^ *PDR1* regulates multidrug transporter gene *CDR1*, which is linked to fluconazole resistance^18^. Using RT-PCR, we showed that *PDR1* and *CDR1* expression were significantly higher in L4 than in other L strains [Figure 4]. To show that G346C affects azole MICs, we first deleted *PDR1* in strains L4 and BG2, then engineered either a *PDR1* G346C mutation or wild-type *PDR1* in these backgrounds. Azole MICs were higher against the engineered *PDR1* G346C strains (256 and 64 μg/mL against L4 and BG2, respectively) than against respective strains with wild-type *PDR1* (32 and 16 μg/mL, respectively) [Supplemental Table 3]. L8 did not carry a *PDR1* mutation, but *CDR1* expression was increased by >12-fold compared with the index strain L1.

Two of 10 strains from the initial positive blood culture were fluconazole-resistant. Blood cultures ~48 hours later, after institution of fluconazole, were fluconazole-resistant. Resistant strains harbored the *PDR1* G346C mutation described above.

#### Patient J.

Patient J was treated with caspofungin for *C. glabrata* BSI, after which he was placed on voriconazole prophylaxis. Forty-seven days after BSI, he developed intra-abdominal infection due to fluconazole-resistant *C. glabrata* SCV (strain J11).

**Figure 5.**
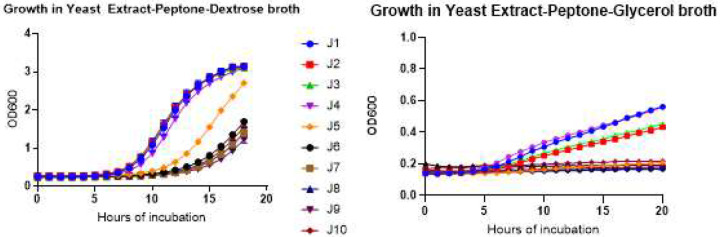

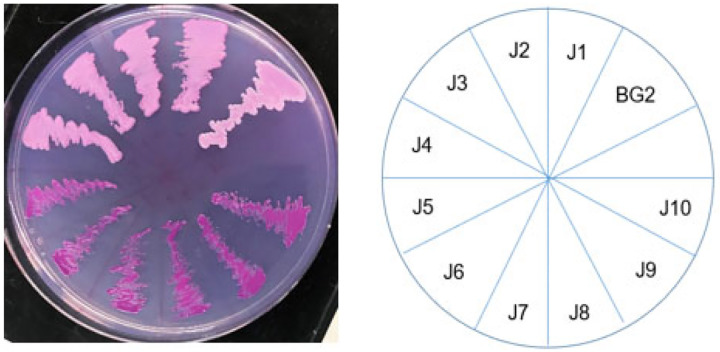

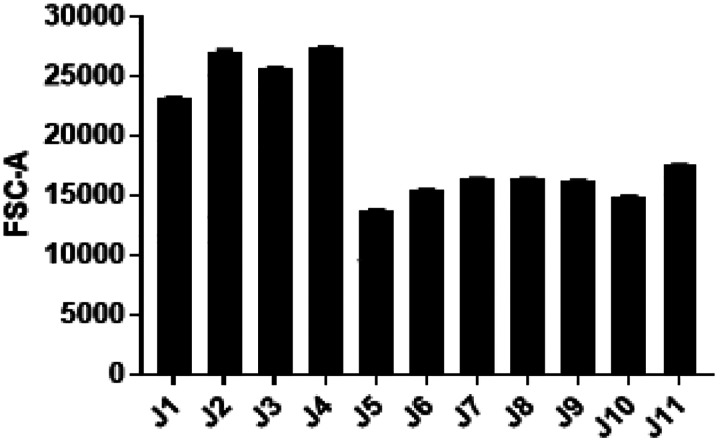

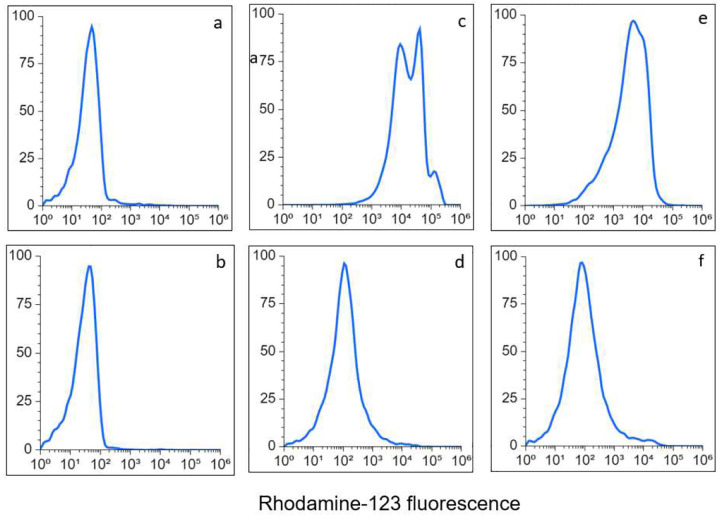

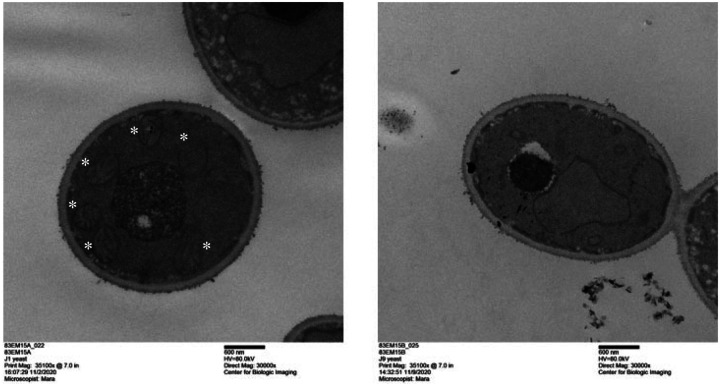

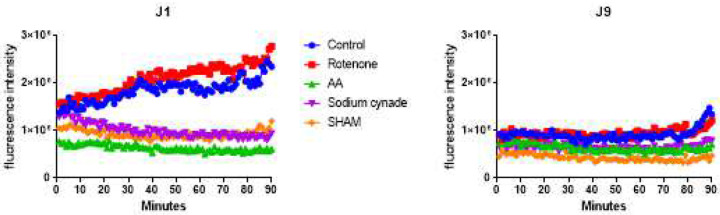
Phenotypes of normal colony variant and small colony variant J strains **5A. Growth rates of J strains in Yeast Extract-Peptone-Dextrose (YPD, left panel) and Yeast Extract-Peptone-glycerol (YP-glycerol, right panel)**. Cells were incubated in respective media at 30°C. Growth of strain J5 is intermediate to that of strains J1-J4 (normal colony variants) and J6-J10 (small colony variants). Note the difference in Y-axis scales for both graphs **5B. Morphology of J strains on eosin plate**. Strain identification is listed on the pie chart on the right. Normal colony variant strains were light pink, whereas small colony variant strains were dark pink to purple. Strains J1-J4 and J5-J10 stain as normal and small colony variants, respectively. Control strain *C. glabrata* BG2 stains as a large colony variant. **5C. Cell sizes of J strains, as measured by FACs**. Cells (1×10^6^ CFU/ml) were grown in 4 ml of YPD broth at 30°C for 24 hours. Strains J1-J4 and J5-J10 demonstrate larger and smaller cell diameters, respectively. **5D. Flow cytometric analysis of rhodamine 123-stained J1 (upper panel) and J9 (lower panel) strains**. Cells (1×10^6^ CFU/ml) were grown in 4 ml of YPD broth overnight at 30°C, then treated with 1 mM sodium azide before rhodamine 123 staining. Unstained cells (a and b) are presented as controls. Stained cells for J1 and J9 cells, before and after treatment with sodium azide, are presented in c and d, and e and f, respectively. X-axis denotes rhodamine-123 fluorescence intensity. The histogram bins are normalized to peak, and the Y-axis denotes percentage of maximal value. Under routine growth conditions, J1 cells exhibited greater fluorescence than did control J9 cells, consistent with higher mitochondrial activity. Intensity of staining of J1 was reduced by roughly 50% following inhibition of electron transport with 1 mM sodium azide. In contrast, sodium azide did not reduce staining of respiratory-deficient strain J9. **5E. Transmission electron micrographs of strains J1 and J9**. Representative images shown here highlight reduced numbers and aberrant morphology of mitochondria in small colony variant strain J9, compared to normal colony variant J1. Several normal appearing mitochondria are denoted by white asterisks (*). **5F. Evaluation of respiratory status of strains J1 and J9 using electron transport inhibitors**. Cells grown in synthetic completed medium for 48 hours at 30°C were untreated or exposed to sodium cyanide 100mM, salicylhydroxamic acid (SHAM) 100 mM or antimycin 1 μM. Oxygen consumption was measured as phosphorescent probe signal intensities (y-axis) *versus* time (x-axis). In absence of drug exposure, oxygen consumption by strain J1 was greater than that by strain J9. Each of the electron transport inhibitors resulted in significant reduction in J1 oxygen consumption, without impacting consumption by respiratory-deficient strain J9.

Strains J1-J4 were NCV in size and morphology on YPD and RPMI agar plates, and in growth rates in YPD broth at 37°C. Strains J6-J10 and J11 were consistent with SCVs (i.e., respiration-deficient *Candida* petite mutants by a constellation of phenotypes, including defective growth in YPD liquid medium, inability to grow on YP-glycerol medium, and deep-violet color on agar containing eosin Y) [Figure 5A and 5B].^19^ Strain J5, which was identified as forming NCV at 48 hours on SDA agar, was intermediate to clones J6-J10 and J1-J4 in growth rate in liquid YPD medium. By flow cytometry, cells of strain J5 were comparable in size to those of strains J6-J10 following growth in liquid YPD, and significantly smaller than those of strains J1-J4 [Figure 5C]. J5 also resembled SCV strains J6-J10 in inability to grow on YP-glycerol medium, and in deep-violet color on eosin Y and trypan blue indicator plate. Therefore, on balance, strain J5 was most consistent with a SCV.

We used GENOME-STRip to estimate mitochondrial genome copy numbers (mtN) of J strains from mapped short reads. Estimated mtN ranged from 1 to 35. Most SCV strains had ≤18 copies (J9, n=1; J5, n=8; J10, n=13; J7 and J8, n=18). In contrast, estimated mtN for strains J1–4 and J6 were 30 and 35, respectively. The average mtN for strains from other patients was 28 (range: 24–31 copies). We further estimated mtN using quantitative PCR of NCV strain J1 and SCV strains J9 and J11. Estimated mtN was significantly higher for J1 than for J9 and J11 (mean 18.4±2.9 vs 0.4±0.1 and 0.3±0.1, respectively; p=0.001, ANOVA).

J1 had significantly greater mitochondrial staining with rhodamine-123, as evident by FACS sorting, than did J9 [Figure 5D]. Electron microscopic images of J1 and J9 corroborated striking differences in numbers of mitochondria per-cell [Figure 5E]. To assess respiration in greater detail, we exposed strains J1 and J9 to various mitochondrial electron transport chain inhibitors. As expected, oxygen consumption by strain J1 was significantly reduced upon exposure to antimycin A, sodium cyanide or salicylhydroxamic acid (SHAM) [Figure 5F]. In contrast, oxygen consumption by strain J9 was already diminished in the absence of electron transport inhibition, and it was not reduced upon exposure to antimycin A, sodium cyanide or SHAM.

In previous reports, respiration-deficient *C. albicans* petite mutants induced in mice during *in vivo* passage experiments or resulting from disruption of Mip1 DNA polymerase were relatively resistant to phagocytosis.^20^ We assayed phagocytosis and killing of strains J1, J5 and J9 by freshly harvested human neutrophils. SCV strains J5 and J9 were more resistant than J1 to neutrophil phagocytosis (12.6±2.7% and 10.9±2.6%, respectively, *vs*. 31.8 ±1.8; *p*<0.0001) and killing (18.5±8.9% and 22.0±2.4% *vs* 39.4.8±7.7; *p*=0.0007 and 0.0001, respectively). J9 was also more resistant to paraquat (survival rates of 77.0±12.1% at 100mM and 15.5±7.4% at 165mM), a redox-active drug that generates endogenous superoxide anions, than J1 (survival rates of 33.4±12.8% at 100mM and 1.4±0.7% at 165 mM; p=0.0005 and 0.02, respectively)

Studies have shown that dysfunctional mitochondria activate *PDR1* and, in turn, *CDR1*.^21^ As expected from these reports, *PDR1* and *CDR1* were up-regulated by 14.6±4.0-fold and 257±11.8-fold, respectively, in strain J9 compared with strain J1 (RT-PCR). Along these lines, fluconazole and voriconazole MICs were higher against strains J6-J10 (≥ 64 μg/mL and 16 μg/mL, respectively) than they were against J1-J4 (4–8 μg/mL and 0.5 μg/mL, respectively). Fluconazole and voriconazole MICs against J5 (32 μg/mL and 1 μg/mL, respectively) were intermediate to those against the other strains.

**Figure 6.**
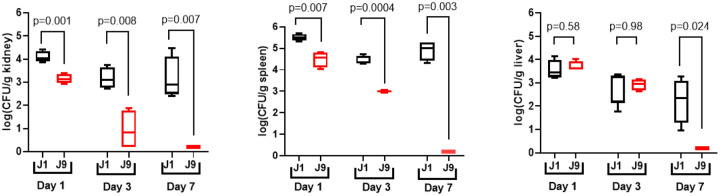
Tissue burdens of *C. glabrata* strains J1 and J9 in kidneys, spleens and livers of mice during hematogenously disseminated infections. Mice were infected intravenously (IV) with 1.5 × 10^8^ CFU of respective strains, and tissue were sacrificed for CFU enumeration at days 1, 3 and 7. Log_10_ tissue burden per organ are presented in box and whisker plot below. Strain J9 was significantly attenuated for virulence in all target organs. P-values (two-tailed) for pairwise comparison were determined using student’s t tests with Welch’s correction. For kidney tissue burden, Welch-corrected t and degree of freedom were 5.98 and 5.76 at day 1, respectively; 4.58 and 4.51 at day 3, and 6.54 and 3.0 at day 7, respectively. The corresponding date for spleen were 5.03 and 4.06 at day 1, 13.51 and 3.45 at day 3 and 20.32 and 3.0 at day 7 and for liver were 0.59 and 4.56 at day 1, 0.03 and 3.59 at day 3, and 4.26 and 3 at day 7.

Finally, we compared strains J1 and J9 for virulence during hematogenously disseminated infections of mice. None of the mice died at day 21 following lateral tail vein injections of 10^7^ CFU of either strain. J9 caused significantly lower tissues burdens than J1 in kidneys and spleens at 1, 3 and 7 days, and in livers at 7 days [Figure 6]

#### WGS and phenotypic characterization of C. glabrata strain J11.

**Figure 7.**
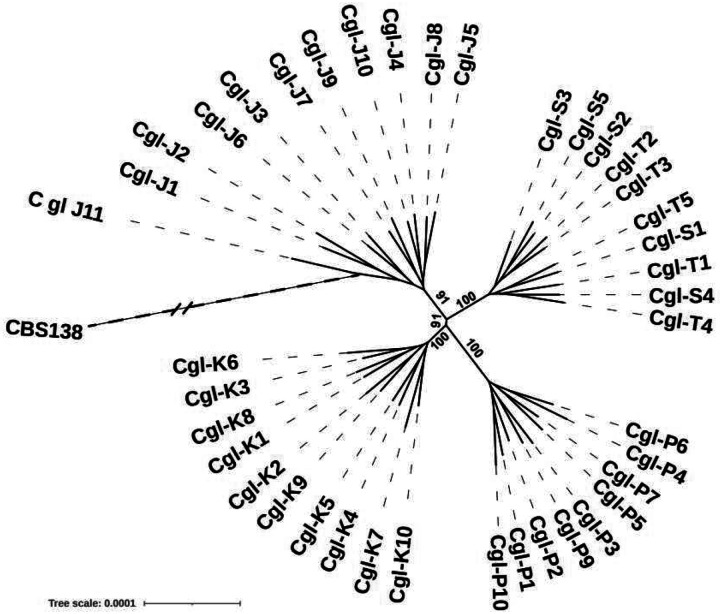

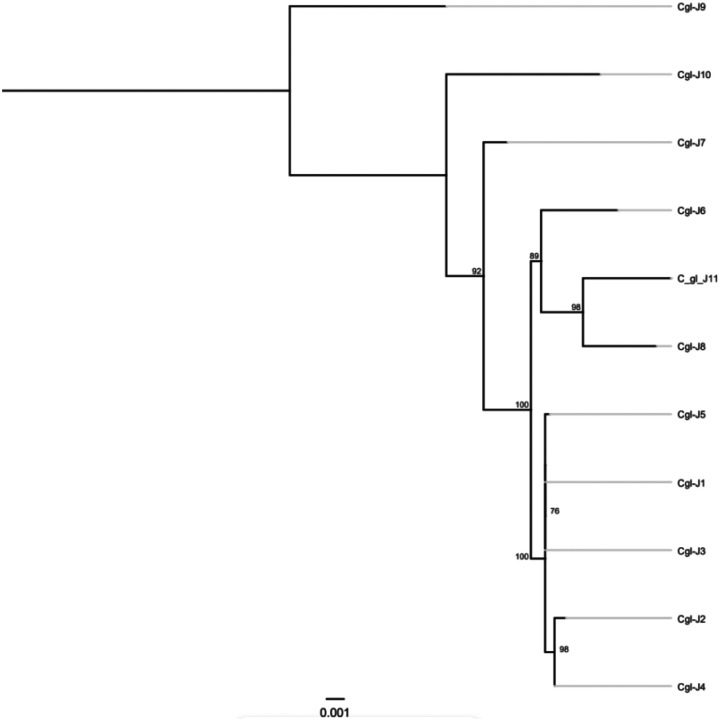
Phylogenetic analysis of ST3 *C. glabrata* strains, including relapsing infection strain J11. **7A. Radial phylogenetic tree of the nuclear genomes of all strains belonging to ST3 from patients J, K, P and ST**. Note that J11 clustered with the 10 BSI strains from patient J (J1-J10). Other strains clustered by patient. **7B. Phylogenetic tree of mitochondrial genome of the 11 J strains**. Note the NCV strains (bottom 5 strains, J1-J5) cluster with each other and the SCV strains (top 6 strains, J6-J10 and the J11) cluster with each other. J11 is closest to J8 by mitochondrial genome.

Strain J11 was recovered by the clinical microbiology lab from a culture of an intra-abdominal abscess 47 days after the index BSI. J11 was a SCV that demonstrated slow growth in YPD media, inability to grow in YP glycerol, small size on SDA plates, purple colonies on eosin plates, and resistance to fluconazole, voriconazole and posaconazole. J11 clustered with other J strains on nuclear genome phylogenetic tree [Figure 7A]. Therefore, J11 was highly related to J strains recovered from the initial blood culture. On mitochondrial phylogenetic tree, strain J11 clustered with SCV strains, and was closest to strain J8 [Figure 7B].

#### WGS and phenotypic characterization of C. glabrata recovered from blood culture spiked with strain J1.

To assess if *C. glabrata* genotypic and phenotypic diversity might arise during growth *in vitro*, we spiked a sterile blood culture bottle with index strain J1. After 3 days of incubation, turbidity was observed. Aliquots sub-cultured on SDA plates revealed homogenous colonies consistent with NCVs. There was no evidence of SCVs after 5 days of incubation. Ten strains from randomly selected colonies had growth rates and susceptibility to fluconazole, voriconazole and posaconazole that were similar to those of strain J1. Strains obtained from spiked blood culture demonstrated significantly fewer nucleotide differences than strains J1-J10 (p<0.0001 for both nuclear and mitochondrial genomes).

## DISCUSSION

To our knowledge, this is the first study to demonstrate genotypic and phenotypic diversity of Candida in blood cultures of individual patients with BSI. We showed that positive blood cultures from each of 10 patients harbored clonal but genetically diverse *C. glabrata* strains that differed by SNPs and, to a lesser extent, insertions, deletions, and presence or absence of specific genes. In 2 patients, genetically distinct strains exhibited unique phenotypes that were potentially important under antifungal selection pressure and during BSIs, including differences in susceptibility to antifungal agents and phagocytosis. In both patients, blood cultures were comprised of mixed fluconazole-susceptible and -resistant populations, but the clinical microbiology laboratory only identified a fluconazole-susceptible index strain. Diversity in drug susceptibility was likely clinically relevant, as fluconazole-resistant *C. glabrata* strains were subsequently recovered by the clinical laboratory during persistent BSI (patient L) or relapsing invasive candidiasis (patient J). In one patient (J), fluconazole-resistant strains were respiration-deficient SCVs, which carried mitochondrial genome mutations that were not present in fluconazole-susceptible, NCV strains. SCVs were also not identified by the clinical laboratory during the initial BSI. Taken together, our data challenge the long-standing, “single organism” model of pathogenesis, and suggest a new population-based paradigm of *C. glabrata* genotypic and phenotypic diversity during BSIs. If validated in other studies, results have potential implications for medical and clinical microbiology practices, and for understanding emergence of *C. glabrata* antifungal resistance, treatment responses, pathogenesis and adaptation.

Most studies of Candida diversity in patients have characterized longitudinal, rather than contemporaneous strains. WGSs of *C. glabrata* or *C. albicans* strains from serial oral, vaginal, blood, stool and respiratory cultures revealed within-patient differences in SNPs, indels, gene CNVs, aneuploidies, and loss of heterozygosity (LOH, for *C. albicans*)^5,7,22–26^. Longitudinal emergence of antifungal resistance, often associated with appearance of resistance-conferring mutations, is well-recognized among *C. glabrata*.^26,27^ In contrast, there are few studies of Candida diversity from a single site at a given timepoint. In a *Candida auris* outbreak investigation, probabilistic analysis of WGS data from 6–12 pooled colonies suggested mixed colonization or disease in ~25% of patients^28^; it is unclear if heterogenous populations were identified from blood cultures. In another study, WGS of strains from 3 independent *C. albicans* colonies from oral cultures of healthy volunteers clearly demonstrated that each strain was unique, mostly due to SNPs and short-range LOH^29^. Finally, in a multi-center study, 5.6% and 8.1% of positive Candida cultures from blood and any clinical sites, respectively, had antifungal polyresistance, defined as heterogenous susceptibility testing results among 5 independent colonies^30^. Such mixed populations were detected in 15.3% of *C. glabrata*-positive clinical samples, a frequency in keeping with our description of unrecognized azole-resistant strains in 2 of 10 patients. Contemporaneous strains in the multicenter study did not differ by multilocus sequence type, but WGS was not performed.

*C. glabrata* and most pathogenic *Candida* spp. are GI tract commensals. Genotypically and phenotypically diverse bacterial populations are increasingly recognized during colonization and chronic infections of non-sterile sites, including GI tract, lungs, and skin^13–15^. Aside from our recent demonstration that positive blood cultures of patients with carbapenem-resistant *K. pneumoniae* BSIs were comprised of genetically variant, clonal strains that differed in antibiotic susceptibility and virulence^16^, there are scant data on microbial diversity during acute infections of putatively sterile sites.^31^ Numbers of nucleotide differences (i.e., SNPs, indels) between contemporaneous *C. glabrata* BSI strains here (pairwise average per-patient: ~3,500) were broadly comparable to those previously reported among contemporaneous *C. albicans* from oral cultures (~500–5,100 SNPs)^29^. Mutations in our strains were predominantly synonymous and found in non-coding regions. Similar findings in a previous study of *C. albicans* passed *in vitro* and *in vivo* were felt to reflect purifying selection that limited accumulation of mutations in protein-coding sequences^3^. More recently, however, investigators were surprised to find enrichment of non-synonymous, coding sequence mutations among serial *C. glabrata* BSI strains^26^. Reasons for discrepancies between studies are unclear, and merit further exploration.

In keeping with previous data for *C. glabrata*, we found that genes encoding adhesins and other cell wall proteins were over-represented as sites of non-synonymous mutations^3,7,8^. Such results were not surprising since repeat-rich, subtelomeric regions that are home to *EPA* and other prominent adhesin gene families are hotspots for chromosomal mutations^3,7,8^. These results suggest potential functional implications for interactions with the host. Although expansions of adhesin genes like those of the *EPA* family are common in *C. glabrata*^32,33^, CNVs were infrequent among contemporaneous strains here. Moreover, large-scale, structural genome variations like aneuploidies or polyploidies were not detected among strains in our patients. Although these large scale chromosomal changes have been linked to antifungal resistance in some longitudinal clinical Candida strains^7,34^, several studies have reported that this phenomenon was relatively uncommon^3,29^.

Azole resistance in various *C. glabrata* strains from patients L and J was associated with increased expression of transcriptional regulator gene *PDR1* and, in turn, up-regulation of efflux gene *CDR1*. Azole-resistant strains from patient L harbored a previously uncharacterized G346C mutation in the *PDR1* central regulatory domain^17^. Using site-directed mutagenesis, we demonstrated that G346C is a gain-of-function mutation that results in *PDR1* hyperactivation and enhanced downstream *CDR1* activation. Azole-resistant strains from patient J carried wild-type *PDR1*, but they exhibited SCV phenotypes including slow growth on enriched medium, attenuated proliferation in non-fermentable carbon sources, and mitochondrial and aerobic respiratory defects. Mitochondrial derangements have been shown to activate the pleiotropic drug resistance Pdr pathway that globally upregulates expression of drug efflux pump genes (e.g., *CDR1*), leading to azole resistance^17,20,21,35,36^. The greatest azole resistance and strongest SCV phenotypes were in strains with the most SNPs in mitochondrial genes, regardless of whether they had normal (J6) or reduced (J7-J10) numbers of mitochondria. NCV strains J1-J4 had normal numbers of mitochondria and few mitochondrial gene mutations. SCVs were likely missed by the clinical laboratory because they were not visualized on blood or SDA agar plates until ≥84 hours after sub-culture. Therefore, SCV prevalence may be under-estimated unless culture plates are assiduously monitored for several days after Candida growth is detected. Our finding has clinical significance since patient J subsequently had a relapsing infection due to a fluconazole-resistant SCV strain (J11) that clustered phylogenetically with SCVs from the initial blood culture.

Mitochondria possess their own genetic material that evolves independently from the nuclear genome. In certain *C. glabrata* strains, the mitochondrial genome is hyper-diverse compared to the nuclear genome^26^. On the whole, we found that mitochondrial genome phylogeny was less sensitive than WGS phylogeny in identifying within-patient strain diversity. Nevertheless, data for J strains indicate that mitochondrial genome analysis may be a useful complement to WGS phylogeny for *C. glabrata* with apparent respiration deficiencies, SCV phenotypes, growth defects or decreased antifungal susceptibility. It is unclear what precipitated mitochondrial dysfunction and emergence of SCVs among J strains. Azole exposure has been linked to mitochondrial damage^37–41^, and patient J received 28 days of fluconazole prophylaxis prior to BSI. In fact, the relatively few clinical *C. glabrata* SCV strains reported to date were recovered from azole-experienced patients.^38,42^ Alternatively, mitochondrial damage could have stemmed from oxidative stress from host phagocytes.^20^ Regardless of mechanism, dysfunctional mitochondria activate compensatory responses that confer adaptive advantages with cross-resistance to both azole and phagocytic killing^20,21,35^. Compared to NCV strain J1, SCV strain J9 was relatively resistant to neutrophil phagocytosis and killing, but it was significantly attenuated for virulence in mice with hematogenously disseminated candidiasis. Azole resistance in SCV strains was associated with growth defects under various conditions in absence of drug exposure, which likely offset potential advantages afforded by evasion of phagocytosis.

Previous studies of virulence of *C. glabrata* SCVs have yielded conflicting results. In one report, an SCV generated by ethidium bromide treatment exhibited reduced virulence during murine disseminated candidiasis.^43^ In another study, however, a fluconazole-resistant oropharyngeal *C. glabrata* SCV was more virulent than an antecedent fluconazole-susceptible strain^38^. We previously reported that a *C. albicans* SCV that emerged after hematogenous passage through mouse organs caused lower mortality and acute tissue burdens during disseminated candidiasis than its parent strain, but it persisted within tissue for a prolonged period^19^. Results are difficult to compare between studies since strains were not isogenic and they were created through different methods. Moreover, virulence is a complex and multi-factorial phenomenon, which can vary based on site of infection and conditions within a given host. For example, SCV strain J9 was less virulent than NCV J1 following intravenous inoculation in our mouse model, but nevertheless SCVs were able to persist in patient J after resolution of candidemia and re-emerge to cause relapsing infection. The concept of virulence is particularly complex for an opportunistic pathogen like *C. glabrata* that has significant redundancy in virulence determinants, lacks a dominant virulence factor, and often causes diseases in patients with immunodeficiencies and other host defense impairments.

A strength of our study design is that *C. glabrata* were collected as blood cultures were being processed according to standard clinical microbiology lab practices. We believe that genotypic and phenotypic diversity emerged at sites of colonization such as the GI tract prior to bloodstream inoculation. While mutations occurring during blood culture incubation may have also contributed to within-patient differences among strains, these are unlikely to account for the extent of diversity we observed in each of our 10 patients. Indeed, incubation of index strain J1 in a sterile blood culture bottle resulted in significantly fewer nucleotide differences among recovered strains than were observed among J1-J10 (p<0.0001), and it did not lead to emergence of SCVs. In a WGS analyses of *C. glabrata* from longitudinal cultures of various body sites over 0–90 days, investigators previously concluded that inter-strain genetic variation was primarily due to standing, pre-existing diversity within the population rather than to accumulation of new mutations^7^. It is unclear whether within-patient diversity may have stemmed from one-time inoculation of the blood with a mixed population of strains, serial inoculation of strains, and/or mutations emerging within the bloodstream prior to collection of blood cultures.

A limitation of this study was that we sequenced only 10 strains per blood culture bottle, which might not adequately represent the entire *C. glabrata* population. In the future, metagenomic sequencing performed directly on DNA recovered from the bloodstream might afford more comprehensive coverage of microbial variants. At present, this approach is limited by relatively low concentrations of microbial DNA, and challenges in assigning sequence variations to individual strains^16^. We only sequenced strains from the first episode of BSI. Future studies of longitudinal samples may provide insights into changes in populations, strain evolution, and importance of pre-existing *vs. de novo* antifungal resistance. Our results cannot be extrapolated to BSIs by other Candida spp. or other pathogens. The extent of diversity we detected may reflect particular features of our patients, who were severely ill and who had extensive past medical histories (Table 1). Six patients had polymicrobial BSIs that included pathogens in addition to *C. glabrata*. As such, diversity among *C. glabrata* in the blood was not surprising. Moreover, 5 patients had recent antifungal exposure or breakthrough BSIs while receiving an antifungal agent, and 2 patients had invasive candidiasis within the previous 90 days. Data here and from our earlier investigation of carbapenem resistant *K. pneumoniae* suggest that genotypic and phenotypic microbial diversity may be especially relevant to BSIs by enteric opportunistic pathogens.

In conclusion, we identified genotypic and phenotypic diversity among *C. glabrata* from blood cultures of individual patients. Our findings suggest that *C. glabrata* diversity arising in response to selective pressures during commensalism, including that imposed by antifungal therapy as in several patients here, may result in variant strains that are better able to cause opportunistic infections. Building upon our data for *C. glabrata* and *K. pneumoniae*, follow-up studies are warranted to investigate BSIs by other GI enteric opportunistic pathogens, including different Candida spp., and to compare diversity within the GI tract with that encountered during BSIs. A pressing question is whether microbial diversity has broad clinical significance, as implied by the unrecognized antifungal resistance in patients L and J. Specific issues for further study, in addition to those raised above, include defining how often clinical laboratories fail to identify antifungal resistant variants (i.e., heteroresistance), whether such events lead to treatment failures, and the relative importance of pre-existing versus *de novo* resistance. Results of such studies will determine whether clinical laboratory practices and treatment decision-making will need to move beyond the longstanding focus on individual strains, and instead consider microbial populations at sites of infection. In the long term, basic research directed toward understanding how microbial diversity and adaptation facilitate commensalism and pathogenesis of different types of invasive infections may identify new paradigms for preventing and ameliorating disease.

## METHODS

### Clinical strains and growth conditions.

Blood culture bottles from patients with *C. glabrata* BSI were obtained from the University of Pittsburgh Medical Center (UPMC) clinical microbiology laboratory along with the isolate used for species identification and susceptibility testing (“index strains”). We streaked 10 μL from the positive blood culture bottle from each patient onto 2 Sabouraud dextrose agar (SDA) plates and incubated overnight at 35°C. *C. glabrata* were confirmed by matrix assisted laser desorption ionization-time of flight mass spectrometry. A strain isolated from each of 4 to 9 morphologically indistinguishable colonies from each patient underwent Illumina NextSeq whole genome sequencing (WGSing). Index strains underwent both short-read Illumina WGS and Oxford Nanopore (ONT) using MinION sequencing.

### DNA extraction and sequencing.

For short-read sequencing, cells were grown in YPD at 30°C overnight, then harvested and resuspended in 1.2M sorbitol, 50mM EDTA. DTT and lyticase were added to the suspension to 10 mM and 85 U/reaction, respectively. Cells were incubated at 37°C for 45 min to make spheroplasts, which were harvested for genomic DNA extraction using Qiagen’s DNeasy Blood and Tissue Kit (Hilden, Germany). For long-read sequencing, high molecular weight genomic DNA from overnight grown cells was extracted following the protocol of Denis et al^44^. DNA sequencing libraries were prepared with the Nextera XT (Illumina, San Diego, USA) protocol according the manufacturer’s directions. Oxford Nanopore libraries were prepared using the Rapid (Oxford Nanopore, Oxford, UK) protocol according the manufacturer’s directions. Illumina libraries were pooled with unique barcodes and sequenced to at least 50X coverage with 2×150 bp paired end reads. Oxford libraries were pooled with unique barcodes and sequenced to at least 30X coverage.

### Bioinformatic analyses.

Raw read zipped files were analyzed through a pipeline built using NextFlow^44^, which integrates Burrows-Wheeler Aligner (BWA)^45^ for reads mapping to the *C. glabrata* CBS138 genome sequence^46,47 46^, downloaded (on 4/30/2020) from Candida Genome Database^47^, and Genome Analysis Toolkit (GATK)^48^ for deduplicating and sorting of mapped reads, variant calling and filtering after a base quality score recalibration (BQSR). SnpEff was used for variant annotation^49^. VCF variant files from all strains were merged using BCFTOOLS^50^, and fasta aligned sequences for each contig were extracted from the merged VCF file using a python script by Stephan Kamrad (https://github.com/Bahler-Lab/alignment-from-vcf). All aligned contigs were then merged and converted to a phylip format using a perl script (https://github.com/nylander/catfasta2phyml). A phylogenomic tree was built using FastTree^51^ or RaxML^52^. The tree topology was manipulated in Geneious (Biomatters Ltd, Auckland, New Zealand), iTol v6^53^, or Adobe Illustrator (Adobe Inc., San Jose, CA). In addition, we extracted an alignment of sequence type (ST) genes and determined the ST for all strains using PubMLST (https://pubmlst.org/). To analyze genes that vary within patients, we selected variants that were called in one or more strain(s), but not all strains within each patient. We focused on variants leading to non-synonymous amino acid substitutions, disrupted start or stop codon, and frameshift insertions or deletions. The list of genes corresponding to these selected variants were analyzed for enrichment in the Candida Genome Database^47^.

Gene deletions/duplications were analyzed using two strategies. The first employed GENOME-Strip^54^ and was based on coverage depth in addition to information from split reads, paired reads, and the assembly of reads. In the second strategy, we employed a Nextflow pipeline that performs *de novo* assembly of long reads from the representative of each patient’s strains using FLYE^55^. Short reads were mapped to the representative strain’s assembly using BWA, and Pilon^56^ was used for error correction and to polish and produce the final assembly. Assembled genomes within each patient were aligned using progressiveMauve^57^. The alignments were visually inspected in Mauve to detect gene deletions/duplications. To estimate mitochondrial copy number for each strain, we used GSUTILS scripts from GENOME-STRIP, which can estimate a gene/genomic region copy number. All programs were run using the University of Pittsburgh Center for Research Computing resources.

### Reverse transcription-quantitative PCR (qRT-PCR).

RNA was harvested from yeast cells in exponential growth in Yeast Extract-Peptone-Dextrose (YPD) broth using the RiboPure RNA purification kit for yeast (Invitrogen), and then treated with DNaseI. cDNA was synthesized using Verso cDNA synthesis kit (Fisher Scientific). Genomic DNA contamination was checked by PCR with primers flanking the intron of *C. glabrata ACT1*. Primers used are summarized in Supplemental Table 1^43,58,59^. Quantitative PCR with SYBR Green qPCR Master Mix (Fisher Scientific) was performed with 1:10 diluted cDNA. Target gene expression was calculated using the ΔΔCT method, with normalization to the housekeeping genes *ACT1* and Cg18S. All experiments were done in independent biological triplicates and are shown as mean with standard deviation (SD) for each time point.

For mitochondrial copy number determination, we used quantitative PCR, with *COX1* as target for mitochondrial gene and *ACT1* for control (Supplemental Table 1). The amplification was performed using Maxima SYBR Green qPCR Master Mix (ThermoFisher Scientific, Waltham, MA, USA).

### Construction of *PDR1* mutants.

To introduce a G346C mutation, *PDR1* was first disrupted using the SAT1 flipper method.^60^ ~500-bp proximal gene (F1) and distal fragments (F2) of the target region were cloned into pSFS2A. which was linearized by digestion with *Kpn*I and *Sac*I. Deletion cassette (F1-SAT1-F2) was introduced into *C. glabrata* BG2 and L4 cells by electroporation. Correct integration was verified by PCR. SAT1 cassette was recycled by FLP-mediated excision. For *PDR1* replacement, the complete *PDR1* ORF flanked by 500 bp was amplified by PCR from genomic DNA of BG2 (with wild-type (WT) *PDR1*) and L4 (with G346C *PDR1*). PCR products were ligated to pSFS2A-F2. The cloned *PDR1* sequence was confirmed by Sanger sequencing. Resulting plasmids were linearized by *Kpn*I and *Sac*I and transformed into strains BG2 Δ*PDR1* and L4 Δ*PDR1*. All primers used are listed in Supplemental Table 1.

### Transmission Electron Microscopy (TEM).

Microscopy was performed at the University of Pittsburgh Center for Biologic Imaging (CBI). Cells were fixed according to protocols published on the CBI website (https://cbi-pitt.webflow.io/protocols). Specimens were fixed in cold 2.5% glutaraldehyde in 0.01 M phosphate-buffered saline (PBS), pH 7.3, post-fixed in 4% aqueous potassium permanganate, then stained with 2% aqueous uranyl acetate. Cells were dehydrated through a graded series of ethanol, and propylene oxide, and embedded in Poly/Bed^®^ 812. Ultrathin sections (65 nm) were stained with 2% aqueous uranyl and Reynold’s lead citrate and examined on JEOL 1400 Plus transmission electron microscope (JEOL Peabody, MA).

### Flow cytometry.

Respiratory status of cells was investigated by flow cytometry.^61^ Briefly, yeast cells in stationary phase (2×10^6^/mL) were incubated with rhodamine 123 at a final concentration of 10 μg/mL for 30 min at 37°C. To inhibit electron flow of the respiratory chain, cells were pre-incubated with 1 mM sodium azide for 2h before the addition of rhodamine 123. Cell fluorescence was quantified with a FACScan flow cytometer. For cell size measurement, unstained yeast cells were washed and evaluated using FACScan. Forward and side scatter gating (FSC-A and SSC-A, respectively) were recorded for 10,000 events.

### Phenotype assays.

#### Respiration.

Respirometry was carried out using the Oxygen Consumption Rate Assay Kit (Caymanchem) as per manufacturer’s instructions. *C. glabrata* strains were grown in synthetic completed medium 48 h at 30°C. After wash with PBS buffer, 140 μl of yeast (5×10^8^/ml in 1X PBS with 2% glucose) were added to wells of 96-well Costar flat bottom plate containing inhibitors sodium cyanide 100mM, salicylhydroxamic acid (SHAM) 100 mM, or antimycin 1 μM. After overlay with 100 μl oil, plates were measured kinetically with SpectraMax i3x at excitation 380nm, emission 650nm for 90 minutes. Three independent experiments were performed. Phosphorescent oxygen probe signal intensities were plotted versus time.

#### Antifungal susceptibility testing.

Antifungal susceptibility testing was performed using the broth dilution technique according to the Clinical and Laboratory Standards Institute (CLSI) standardized method^62^. Concentrations ranged from 0.25–256 μg/ml for fluconazole, and 0.015–16 μg/ml for voriconazole and posaconozole. For fluconazole, strains were classified as susceptible-dose-dependent (S-DD) or resistant (MIC ≤32 μg/mL and ≥64 μg/ml, respectively) according to CLSI^62^; for purposes of this manuscript, we refer to S-DD as susceptible. Currently there are no defined voriconazole or posaconazole breakpoints for resistance. *C. glabrata* ATCC 90030 was incorporated into each set of experiments as quality control.

#### Adherence assays.

Adherence was assessed using epithelial cells Hep-2 (ATCC CCL23). Yeast cells in exponential phase were added to confluent Hep-2 cells in RPMI 1640 medium without serum at various multiplicities of infection (MOI). Contact between Hep-2 and yeast cells was initiated by brief (1 to 2 min) centrifugation (500 × *g*)^63^. After incubation for 1 hour, nonadherent cells were removed by five washes in PBS. Adherent cells were recovered by lysis of the monolayer using 0.5 mL of 0.1 % triton, 0.5 % SDS, 10 mM EDTA in PBS. Adherent yeast cells were scraped off the well, and serial dilutions were made in distilled water, which were then cultured on YPD agar plates for colony count enumeration. Percentage of adherence was calculated by dividing colony forming units (CFUs) of adherent cells over CFU of input cells and multiplied times 100.

#### Phagocytosis and killing assays.

Phagocytosis and killing assays were performed as previously described with slight modification.^19^ Briefly, fresh polymorphonuclear cells (PMNs) were resuspended in RPMI 1640. Opsonized *C. glabrata* cells (50% normal human serum at 37°C for 30 minutes) were incubated with 10^6^ PMNs at an MOI of 50:1 (PMN:yeast) in 1mL of RPMI 1640 medium containing 5% human serum at 37°C. For phagocytosis, three drops of the sample were cytospun after 15-min of incubation and Gram-stained. Percent phagocytosis was calculated as the proportion of PMNs containing ≥1 blastoconidia after counting 100 PMNs. For killing, PMNs were lysed with sterile water after 2-h of incubation, serial 10-fold dilutions were made, and colony counts were enumerated. The fungicidal activity was calculated as the percent of survival of *C. glabrata* after 2-hour incubation with PMNs. The experiments were performed in triplicate and repeated at least twice.

#### Paraquat killing.

Yeast was grown overnight in Synthetic Complete Medium (SC medium) at 30°C with 250 rpm shaking. Cells were washed twice with 1XPBS and adjusted to 5×10^3^/ml. Paraquat was added to desired concentrations. After 1 hour incubation at 37°C with 750 rpm shaking, serial 10-fold dilutions were made, and colony counts enumerated after 48 h incubation at 30°C.

#### Mouse model of disseminated candidiasis.

CD1 mice (4–6 weeks old) were infected via tail vein injection with 1.5×10^8^ CFU *C. glabrata* strains. Mice were euthanized and liver, kidneys and spleen harvested on days 1, 3 and 7. Organs were homogenized and dilutions plated for total CFU counts on YPD and other selective media (please refer to Results section). Colonies were counted after 48–72 h of incubation at 30°C.

### Statistical analysis.

MICs and CFU/mL were log-transformed prior to statistical analysis. Data are presented as means and standard error for symmetric data and as medians and interquartile ranges (IQR) for asymmetric data. Statistical analyses were performed using GraphPad Prism version 9.4. Student t-test or Mann-Whitney U tests were used for comparisons of 2 groups. Survival curves were calculated according to the Kaplan-Meier method using Prism and compared using Newman-Keuls analysis. For all analyses, *p*<0.05 (two-tailed) was considered significant.

## Supplementary Material

1

## Figures and Tables

**Table 1.** Clinical characteristics of 10 patients with *Candida glabrata* bloodstream infections

Patient	Age Sex	Underlying diseases	Prior invasive candidiasis	Antifungal in the 90 days preceding candida BSI (cumulative duration)	Other positive sterile site culture	Portal of entry	Antifungal MIC μg/mL)	Outcome
C	40 W	Short gut syndrome, chronic TPN	No	No	No	Intravenous catheter CLABSI	CAS, 0.03 FLU, 4 POS, 0.5 VOR, 0.125	Alive
D	58 M	Duodenal perforation, s/p multiple abdominal surgeries and colostomy; malnutrition, on TPN	*C. krusei* IAC *C. albicans* cholangitis, sacral osteomyelitis	CAS (104 days)	No	Intravenous catheter CLABSI	CAS, 0.03 FLU, 4 POS, 0.5 VOR, 0.25	Died day 32
EF	72 M	Esophageal cancer, s/p esophagectomy complicated by trachea-esophageal fistula	No	No	Other cultures, *C. glabrata* and *C. dubliniensis* peritonitis	GI tract	CAS, 0.06 FLU, 64 POS, 2 VOR, 1	Alive
H	64 M	S/p kidney transplant; bladder cancer with obstructive uropathy s/p neobladder creation; intermittent self-catheterization	*C. glabrata* surgical site infection s/p kidney transplant	FLU (23 days)	Polymicrobial BSI, *C.albicans* Other cultures, urine	GU tract Allograft pyelonephritis	CASP, 0.125 FLU, 4 POS, 1 VOR, 0.25	Alive
I	64 M	Esophageal cancer s/p esophagectomy and gastric surgery; anastomotic leak		Breakthrough BSI day 7 of FLU	Polymicrobial BSI, Lactobacillus	GI Mediastinitis and empyema	CAS, 0.125 FLU, 2 POS, 0.5 VOR, 0.25	Alive
J	61 M	Liver transplant with portal vein thrombosis	None	Breakthrough BSI day 28 of FLU	Polymicrobial BSI, Vancomycin-resistant *Enterococcus faecium*	GI/hepatobiliary	CAS, 0.125 FLU, 8 POS, 0.5 VOR, 0.25	Recurrent candidiasis day 47 Died 15 days later
								
K	57 W	Ischemic gut, status post right hemicolectomy	No	No	No	GI	CAS, 0.06 FLU, 16 POS, 0.06 VOR, 0.25	Died day 1
L	81 W	Esophageal dysmotility complicated by esophageal perforation and empyema	No	FLU (16 days)	Polymicrobial BSI, Bifidobacterium Other cultures, *C. glabrata* and *Lactobacillus rhamnosus* pleural fluid	GI Empyema	CAS, 0.125 FLU, 32 POS, 0.125 VOR, 0.25	Died day 13
P	57 W	Pancreatic cancer, biliary obstruction s/p biliary drain	No	No	Polymicrobial BSI, Enterobacter cloacae complex	GI/biliary tract Cholangitis	CAS, 0.125 FLU, 8 POS, 1 VOR, 0.5	Died day 16
ST	27 M	s/p liver transplant 16 years prior with recurrent cirrhosis, s/p TIPS that required revision 1 month earlier	No	No	Polymicrobial BSI, *C. albicans*	Endovascular (TIPS)	CAS, 0.06 FLU, 16 POS, 1 VOR, 0.5	Died day 55

Abbreviations:

Pt, Patient

Age/sex column, W, woman; M, man

Underlying diseases, TIPS, transjugular intrahepatic portosystemic shunt; TPN, total parenteral nutrition

Prior episodes of invasive candidiasis, IAC, intra-abdominal candidiasis

Prior antifungal, CAS, caspofungin; FLU, fluconazole; POS, posaconazole; VOR, voriconazole

Polymicrobial BSI, BSI, bloodstream infection

Portal of entry, CLABSI, central line associated BSI; GI, gastro-intestinal tract

Antifungal MIC (minimum inhibitory concentration as determined by the Clinical Microbiology Lab), POS, posaconazole; VOR, voriconazole

**Table 2.** Within-patient differences in gene copy number variants. Data were analyzed using Nextflow pipeline. Black and gray boxes represent presence and absence of specific genes, respectively. Deletions of 12 genes conferred within-patient differences between strains in 4 patients; 8 of these genes encoded adhesins. We did not observe any within-patient gene duplication.

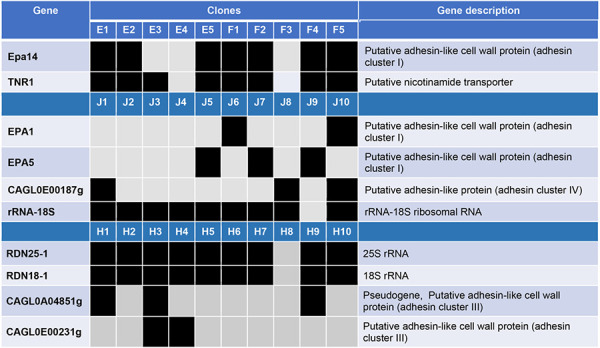 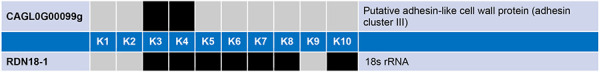

**Table 3.** Biological processes enriched among gene variants that discriminated between *C. glabrata* strains in each patient.

Patient	GO term	Cluster frequency	Background frequency	Corrected P-value	False discovery rate	Genes
**C**	Adhesion to host	3.2% (4/154)	0.1% (5/5615)	5.73E-06	0.00%	*EPA6, EPA7, EPA1, EPA3, EPA11*
						
**D**	Cell-substrate adhesion	5.4% (5/141)	0.1% (7/5615)	7.23E-05	0.00%	*AWP14, EPA6, EPA7, EPA1, EPA3*
						
**I**	Cell adhesion	4.2% (7/167)	0.3% (19/5615)	0.00029	0.00%	AWP14, EPA1, EPA3, CAGL0I07293g, AWP2, CAGL0K12078g, EPA11
						
**L**	Cell adhesion	9.1% (4/44)	0.1% (6/5615)	4.96E-06	0.00%	*EPA6, EPA7, EPA3, CAGL0K12078g, EPA11*
	Adhesion of symbiont to host	4.5%(2/44)	0.1% (4/5615)	0.036	5.08%	*EPA6, PWP7*
						
**EF**	Cell-substrate adhesion	2.2% (5/223)	0.1% (7/5615)	0.00115	0.00%	*AWP14, EPA6, EPA7, EPA1, CAGL0K12078g*
						
**H**	Adhesion of symbiont to host*	5.4% (3/56)	0.1% (5/5615)	0.002	0.00%	*EPA6, PWP7, AED1*
						
**J**	Mitochondrial translational elongation	5.8% (10/206)	1.1% (63/5615)	1.67E-06	0.00%	*tM(CAU)8mt, tW(UCA)1mt, tT(UGU)4mt, tH(GUG)7mt, tE(UUC)10mt, tM(CAU)9mt, tP(UGG)8mt, tC(GCA)4mt, tL(UAA)4mt, tR(UCU)10mt*
	Mitochondrial translation	5.8% (12/206)	1.1% (63/5612)	0.00081	0.00%	*VAR1, LSU, tM(CAU)8mt, tW(UCA)1mt, tT(UGU)4mt, tH(GUG)7mt, tE(UUC)10mt, tM(CAU)9mt, tP(UGG)8mt, tC(GCA)4mt, tL(UAA)4mt, tR(UCU)10mt*
	Mitochondrial gene expression	5.8% (12/206)	1.5% (82/5612)	0.014	0.80%	*VAR1, LSU, tM(CAU)8mt, tW(UCA)1mt, tT(UGU)4mt, tH(GUG)7mt, tE(UUC)10mt, tM(CAU)9mt, tP(UGG)8mt, tC(GCA)4mt, tL(UAA)4mt, tR(UCU)10mt*
	Intron homing	1.5% (3/206)	0.1% (3/5615)	0.02306	0.86%	*Cgai1, Cgai2, Cgai3*
	Cell substrate adhesion	2.4% (5/206)	1.5% (7/5615)	*0.00055*	0.00%	*AWP14, EPA6, EPA1, EPA3, CAGL0K12078g*
						
**K**	Cell-adhesion	7.4% (7/95)	0.3% (19/5615)	2.19E-06	0.00%	*AWP14, EPA6, EPA1, EPA3, AWP2, CAGL0K12078g, EPA11*
						
**P**	Cell-substrate adhesion	5.1% (5/98	0.1% (7/5612)	5.01E-06	0.00%	*AWP14, EPA6, EPA1, EPA3, CAGL0K12078g*
						
**ST**	Cell adhesion	6.7% (10/129)	0.3% (19/5615)	2.14E-09	0.00%	*AWP14, EPA6, EPA7, EPA1, EPA3, EPA23, AWP2, CAGL0K12078g, EPA11, EPA13*

GO term enrichment for non-synonymous gene mutations that discriminated between within-patient strains
