# Supplementary material for: Genotypic diversity and unrecognized antifungal resistance among populations of Candida glabrata from positive blood cultures
Source: Res Sq. 2023 Apr 3:rs.3.rs-2706400. Preprint. [Version 1] doi: 10.21203/rs.3.rs-2706400/v1 (PMC10104189; doi:10.21203/rs.3.rs-2706400/v1)
Supplement: 1 [file NIHPPRS2706400V1-supplement-1.pdf]

Supplemental tables and figures

Supplemental Table 1. Sequence of primers used in this study

Name	Primers (5'-3')	Experiment	References
ChrG679k-For	ATTACAGGGCCAAGCTAAAC	Gene deletion confirmation	This study
ChrG685k-Rev	CCAGTTCCGCCATCTTATTT	Gene deletion confirmation	This study
ChrM550k-For	AACACCTATGATGCAAGCACAAC	Gene duplication confirmation	This study
ChrM550k-Rev	GAAACTCTGCTGTATGTCCCCAT	Gene duplication confirmation	This study
PDR1F1For_KpnI:	ACGGGGTACC AAAGGGAGTGACAGCGAGAAT	Disruption of PDR1	This study
PDR1F1Rev_ApI	CAAGTCAACGTAGGGCCCACCTGGGAATACAACCCAAA	Disruption of PDR1	This study
PDR1F2For_SacII	TGGGCCATCTTCCGCGGGGGAGATTTGAATGAACTC	Disruption of PDR1	This study
PDR1F2Rev_ScI	CTCATAGATTACACGAGCTCTGTGCTACAGACTGCATTGGA	Disruption of PDR1	This study
PDR1ReinFor_KpnI:	ACGGGGTACCAGCCTCCTATTCCGTGGAAA	Reinsertion of PDR1	This study

PDR1ReinRev_ApaI:	CAAGTCAACGTAGGGCCCTCATAGACATGGGTGCTGTGT	Reinsertion of PDR1	This study
PDR1Up For:	TACCCCATATCGTATTGCCA	Disruption confirmation	This study
PDR1Down Rev:	TCATAGACATGGGTGCTGTGT	Disruption confirmation	This study
pMalRev	TACGACTACATCAATGAAATCCAGACAGTC	Disruption confirmation	This study
SAT1For	GGCATTGACCTCTTCACGTATAAAACTAG	Disruption confirmation	This study
Cgl 18S-SYBR-For	TCGGCACCTTACGAGAAATCA	Gene expression	[58]
Cgl 18S-SYBR-Rev	CGACCATACTCCCCCCAGA	Gene expression	[58]
Cgl ACT1-For	CAAATATATAACAATGGATTCT	Excluding gDNA contamination	This study
Cgl ACT1-Rev	GAGTCCAAAACAATACCGG	Excluding gDNA contamination	This study
CgACT1-SYBR-For	TATTGACAACGGTTCCGG	Gene expression	[43]

CgACT1-SYBR-Rev	TAGAAAGTGTGATGCCAG	Gene expression	[43]
CgCDR1-For	CATACAAGAAACACCAAAGTCGGT	Gene expression	[58]
CgCDR1-Rev	GAGACACGCTTACGTTCACCAC	Gene expression	[58]
CgPDR1-For	TTTGACTCTGTTATGAGCGATTACG	Gene expression	[58]
CgPDR1-Rev	TTCGGATTTTTCTGTGACAATGG	Gene expression	[58]
CgERG11-For	CCACCCATTGCACTCTTTGT	Gene expression	[59]
CgERG11-Rev	AGAACGTGGTAGTCCCTTGG	Gene expression	[59]
mito-FOR	AATTCTGATTAATTTTTGTAGGAGCTAATG	Mitochondrion copy number	This study
mito-REV	GCAATAAATGATCCTACTGATGCTAC	Mitochondrion copy number	This study
Act1-FOR	TGGTCGGTATGGGTCAAAAG	Control copy number	This study
Act1-REV	CGTTGTAGAAAGTGTGATGCC	Control copy number	This study

Supplemental Table 2. Variants associated with non-synonymous mutations that discriminated within-patient strains (i.e., mutations found in at least one strain from a patient but not found in all strains, compared to *C. glabrata* CBS138).
Please refer to EXCEL file

Supplement Table 3. Effect of *PDR1* mutation on fluconazole, voriconazole and posaconazole MICs

Strains	Fluconazole MIC (µg/mL)	Voriconazole MIC (µg/mL)	Posaconazole MIC (µg/mL)
BG2	16	0.25	2
BG2 with ΔCg*PDR1*	4	<0.125	0.5
BG2 with ΔPDR1_Reinsert WT Cg*PDR1*	16	0.5	2
BG2 with ΔPDR1_Reinsert G346C Cg*PDR1*	64	1	2
L4	256	8	8
L4 with ΔPDR1	4	<0.125	0.5
L4 with ΔPDR1_Reinsert WT Cg*PDR1*, strain #1	32	1	2
L4 with ΔPDR1_Reinsert WT Cg*PDR1*, strain #2	32	1	2
L4 with ΔPDR1_Reinsert G346C Cg*PDR1*, strain #1	256	4	>16
L4 with ΔPDR1_Reinsert G346C Cg*PDR1*, strain #2	256	4	>16

Supplementary Figure 1. Sites of within-patient non-synonymous substitutions within *C. glabrata* chromosomes. Substitutions found in some but not all within-patient strains, compared to CBS138 reference strain, were plotted on chromosomes and the mitochondrial genome using the kpPlotRainfall function of the R package karyoploteR. Chromosome ID and coordinates (x-axis) are shown by the gray rectangle (bottom of figure). Black dots show positions of all substitutions, and green diamonds show positions of non-synonymous substitutions. Black and green curves at the top of the figure correspond to density of all substitutions and non-synonymous substitutions, respectively, along the chromosome. Subtelomeric regions tend to accumulate more substitutions including non-synonymous substitutions.

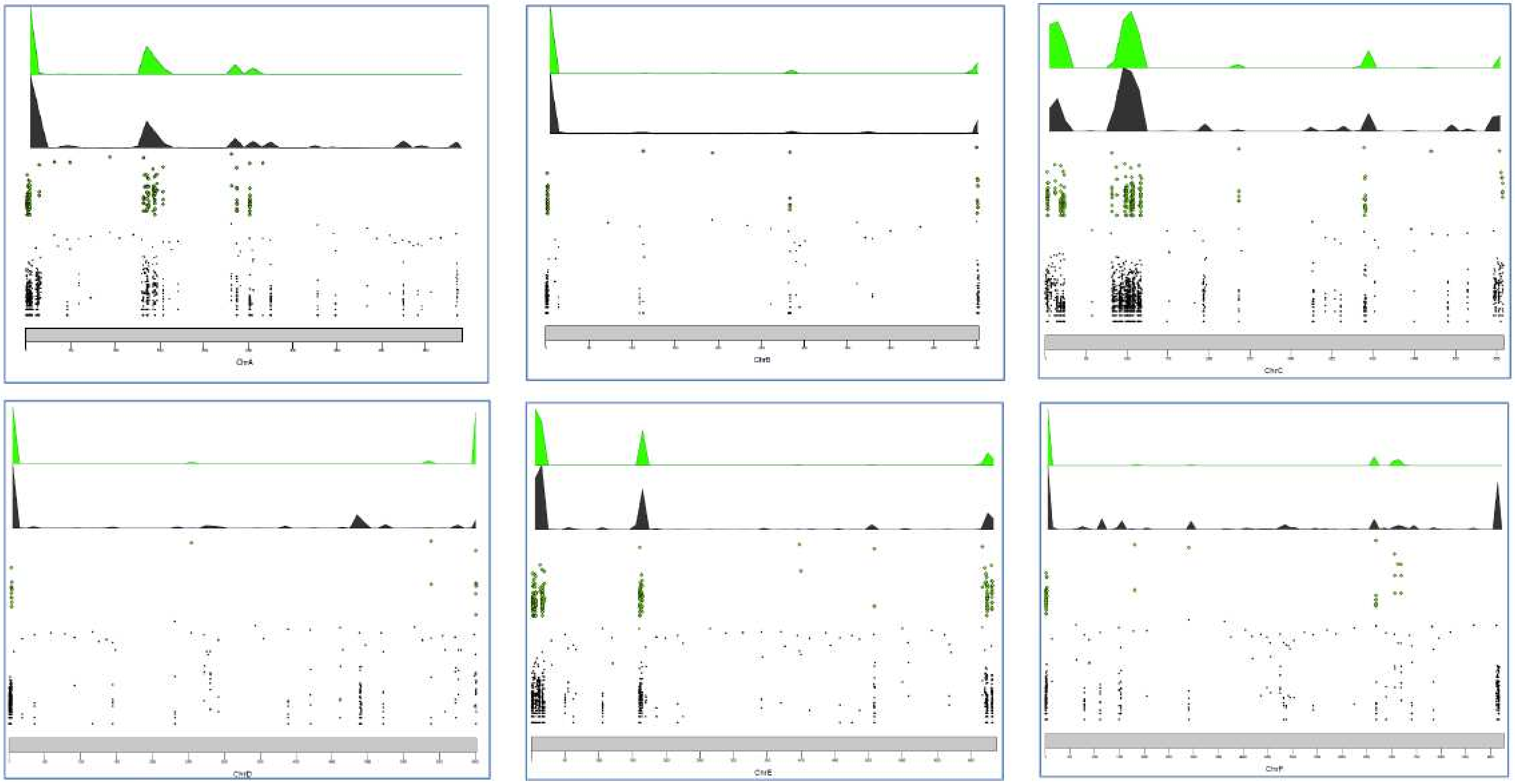

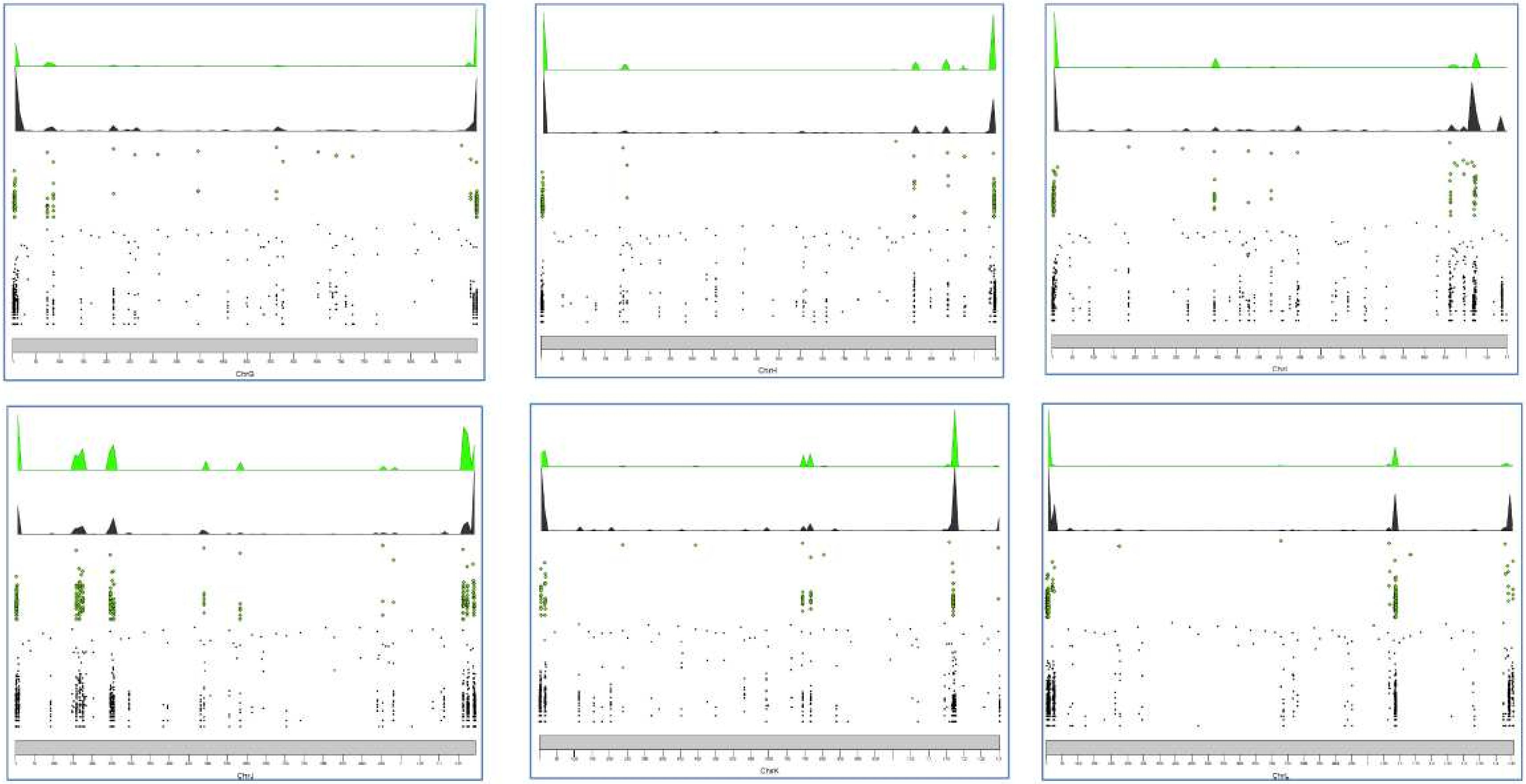

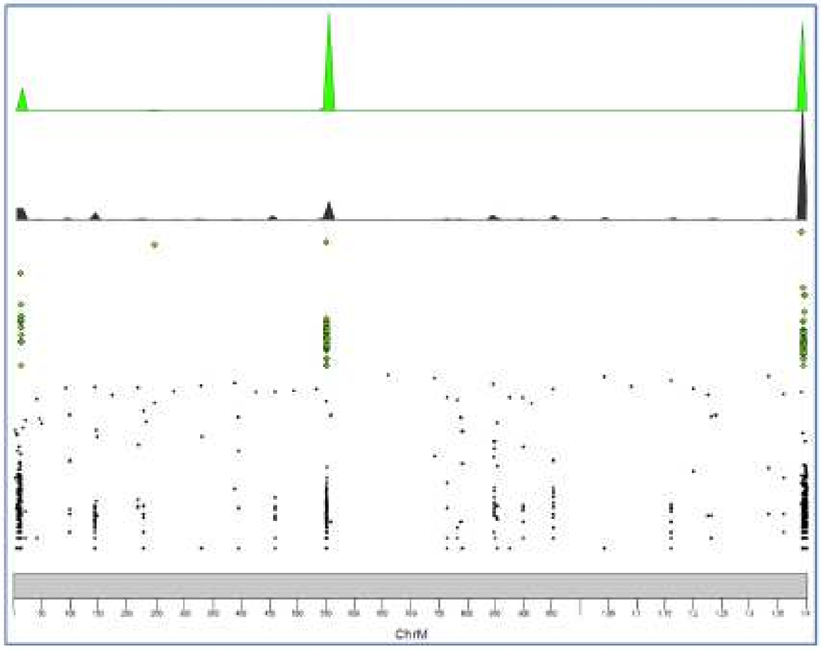
ChrM

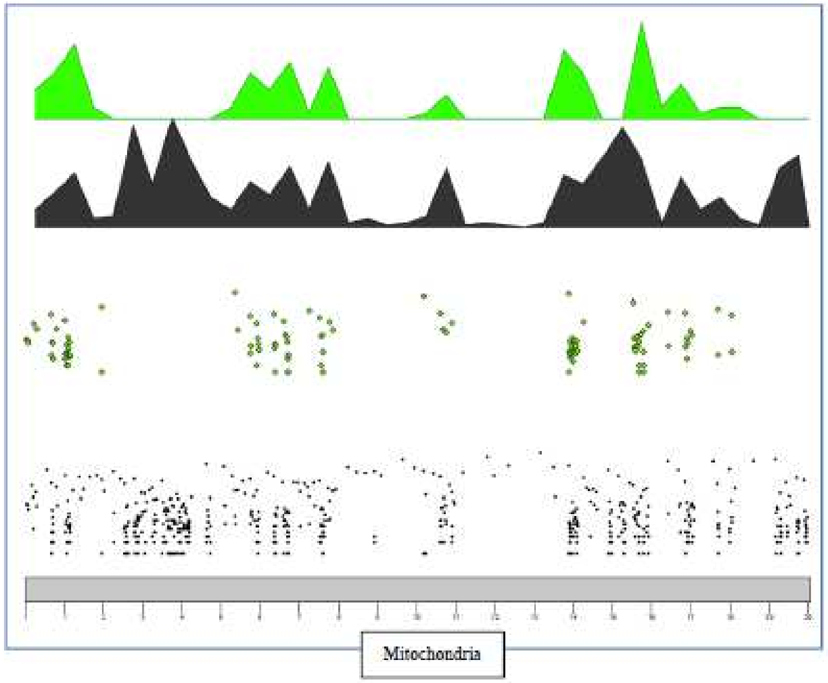
Mitochondria
